# Supplementary material for: Cyclic di-AMP traps proton-coupled K+ transporters of the KUP family in an inward-occluded conformation
Source: Nat Commun. 2023 Jun 21;14:3683. doi: 10.1038/s41467-023-38944-1 (PMC10284832; doi:10.1038/s41467-023-38944-1)
Supplement: Supplementary file 1 — Supplementary Information [file 41467_2023_38944_MOESM1_ESM.pdf]

# Cyclic di-AMP traps proton-coupled $K^+$ transporters of the KUP family in an inward-occluded conformation

Michael F. Fuss[#,1], Jan-Philip Wieferig[#,2], Robin A. Corey[#,3], Yvonne Hellmich[1], Igor Tascón[1,5], Joana S. Sousa[2,6], Phillip J. Stansfeld[4], Janet Vonck*[,2], Inga Hänelt*[,1]

[1] Institute of Biochemistry, Goethe University Frankfurt, Frankfurt am Main, Germany
[2] Department of Structural Biology, Max Planck Institute of Biophysics, Frankfurt am Main, Germany
[3] Department of Biochemistry, University of Oxford, Oxford, UK
[4] School of Life Sciences & Department of Chemistry, University of Warwick, Coventry, CV4 7AL, UK
[5] Current address: Instituto Biofisika (UPV/EHU, CSIC), University of the Basque Country, Leioa, Spain. Ikerbasque, Basque Foundation for Science, Bilbao, Spain
[6] Current address: UCB Pharma, UCB Biopharma UK, Slough, SL1 3WE, UK
[#] These authors contributed equally and may change the order of their names
* Corresponding authors

## Supplementary information

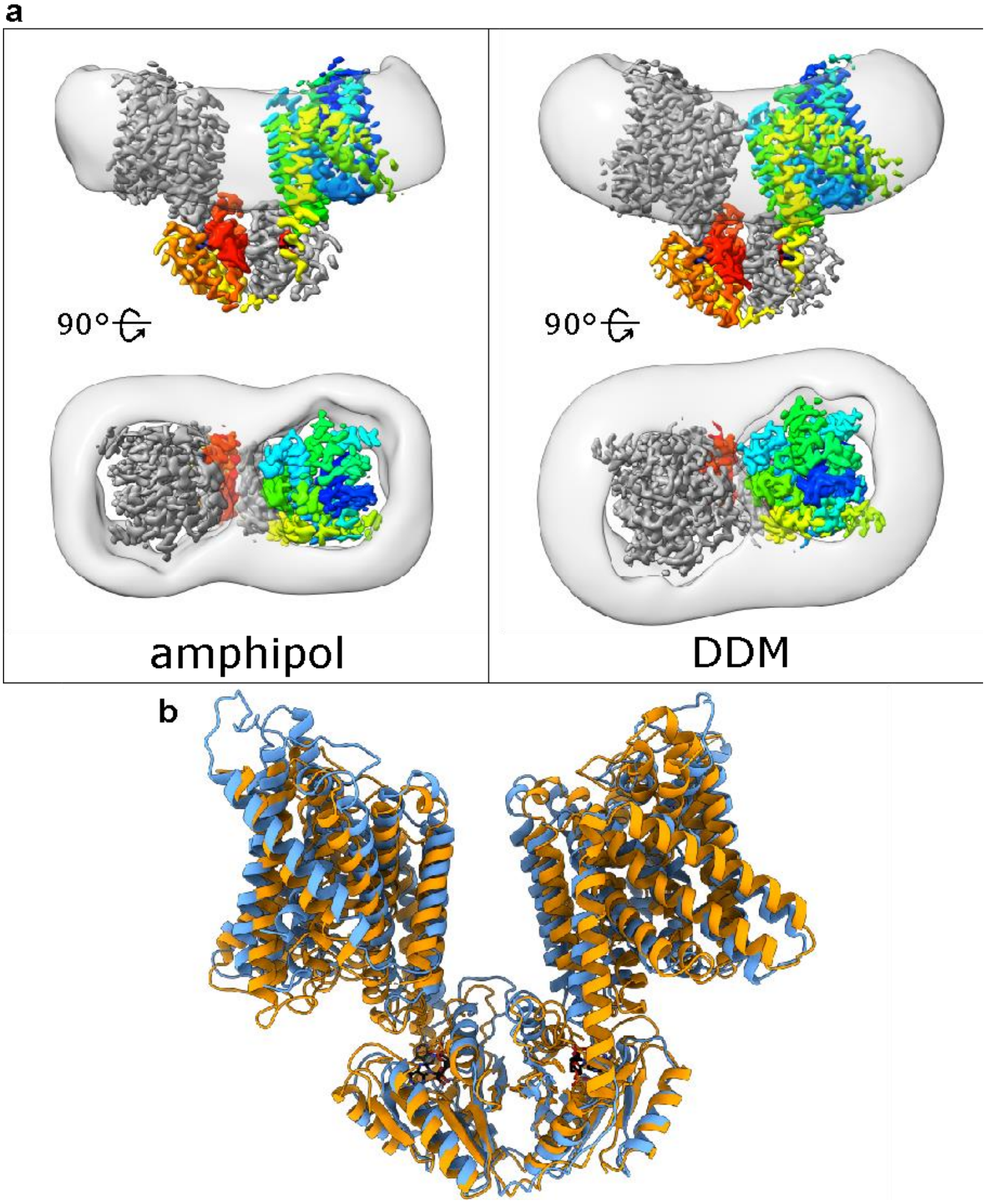


**Supplementary Figure 1: a**) 3.8 Å and 3.3 Å maps of KimA with gaussian filtered ( ʃ = 3) amphipol and DDM belt, respectively. The relaxed, upright dimer architecture of KimA is preserved in the amphipol belt that forms around each TMD (left). In DDM, the TMDs tilt inwards as previously seen in SMALPs. Density is coloured by monomer. One monomer is coloured in a rainbow scheme from N to C terminus. **b**) View of amphipol-reconstituted KimA (orange) aligned on cytoplasmic residues 462 to 606 with the upright dimer as previously predicted using MD simulations[25] (blue). The MD structure was constructed by clustering ca. 6.2 µs of atomistic MD data using gmx cluster with an RMSD cutoff of 0.3 nm. Shown is the state which best represents the cluster for the upright dimer, which accounts for approximately 33% of the total data.

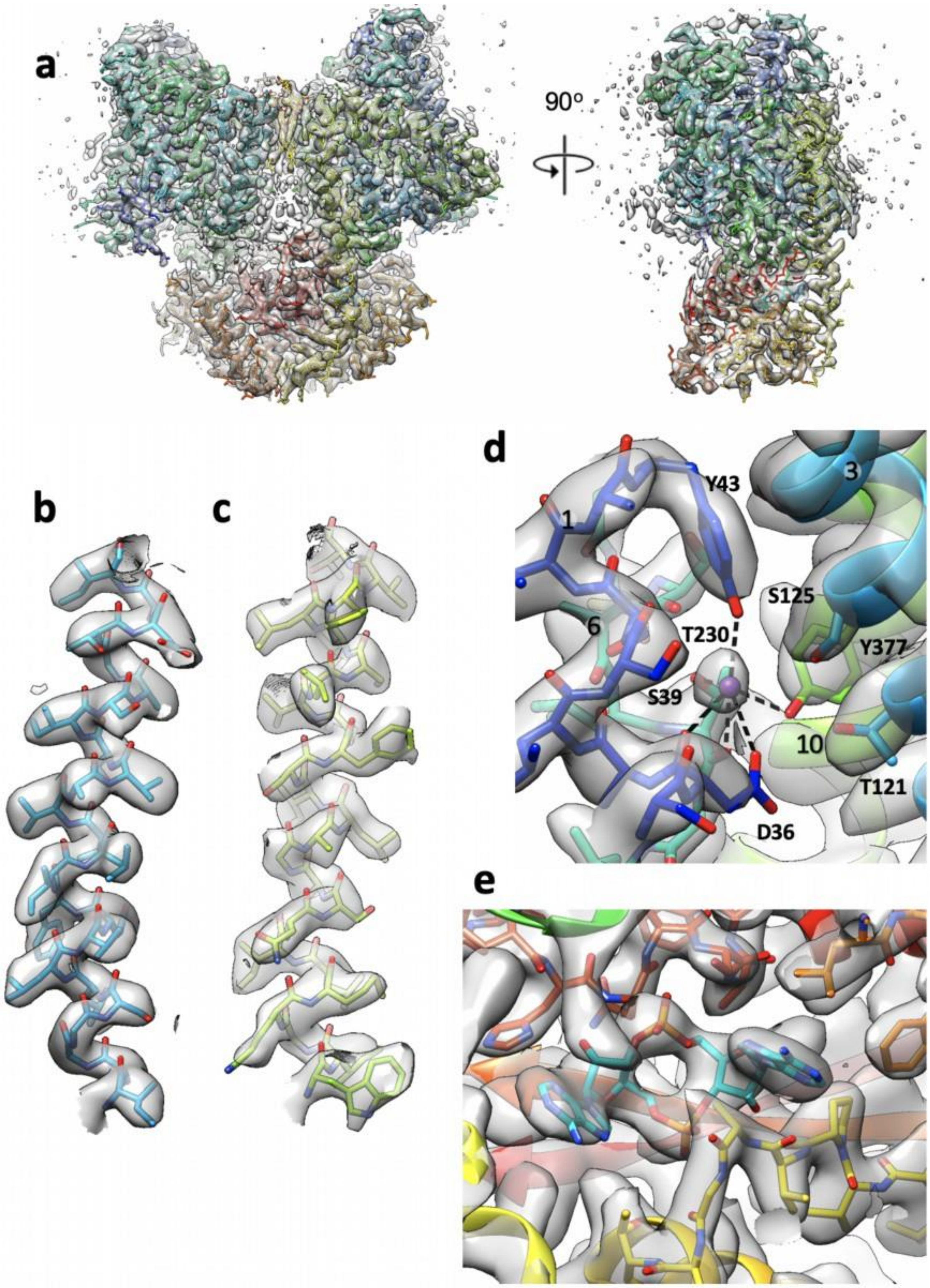


**Supplementary Figure 2**: **3.3 Å cryo-EM map of KimA in DDM. a:** map with fitted model (rainbow colors from blue to red) seen from the front (left) and side (right); **b:** transmembrane helix 109-133 (TM3); c: transmembrane helix 404-428 (TM11); **d:** substrate potassium ion (purple) with coordinating residues; **e:** c-di-AMP binding site. The EM map is shown as transparent surface.

| | | | | | | | | |
|---|---|---|---|---|---|---|---|---|
| No Ligand | Phe568<br>2.06 ± 0.28 | Ile569<br>1.73 ± 0.16 | Trp592<br>1.99 ± 0.41 | Arg587<br>1.79 ± 0.18 | Phe565<br>1.41 ± 0.08 | Ser582<br>1.80 ± 0.21 | Arg337<br>2.5 ± 0.11 | No Ligand |
| | Arg337<br>2.70 ± 0.13 | Ser582<br>1.70 ± 0.13 | Phe565<br>1.56 ± 0.04 | Arg587<br>2.13 ± 0.16 | Trp592<br>2.12 ± 0.18 | Ile569<br>2.10 ± 0.40 | Phe568<br>2.51 ± 0.31 | |

| | | | | | | | | |
|---|---|---|---|---|---|---|---|---|
| Ligand | Phe568<br>1.48 ± 0.02 | Ile569<br>1.62 ± 0.09 | Trp592<br>1.32 ± 0.12 | Arg587<br>1.69 ± 0.08 | Phe565<br>1.30 ± 0.14 | Ser582<br>1.2 ± 0.15 | Arg337<br>1.67 ± 0.14 | Ligand |
| | Arg337<br>1.75 ± 0.15 | Ser582<br>1.29 ± 0.10 | Phe565<br>1.3 ± 0.12 | Arg587<br>1.54 ± 0.40 | Trp592<br>1.32 ± 0.04 | Ile569<br>1.32 ± 0.18 | Phe568<br>1.83 ± 0.21 | |

| | | | | | | | | |
|---|---|---|---|---|---|---|---|---|
| No Ligand | Phe568<br>1.88 ± 0.30 | Ile569<br>1.28 ± 0.05 | Trp592<br>1.22 ± 0.23 | Arg587<br>1.31 ± 0.10 | Phe565<br>0.80 ± 0.02 | Ser582<br>0.90 ± 0.03 | Arg337<br>1.30 ± 0.20 | Ligand |
| | Arg337<br>2.07 ± 0.06 | Ser582<br>1.22 ± 0.14 | Phe565<br>1.25 ± 0.12 | Arg587<br>1.32 ± 0.04 | Trp592<br>0.95 ± 0.04 | Ile569<br>1.28 ± 0.04 | Phe568<br>1.30 ± 0.10 | |

**Supplementary Figure 3**: **Raw RMSF values for the data in Figure 3b, as well as KimA with asymmetric ligand occupancies.** Data in top and middles row are from 3 x 2.2 µs simulations. Data from the bottom row are from 3 x 500 ns simulations.

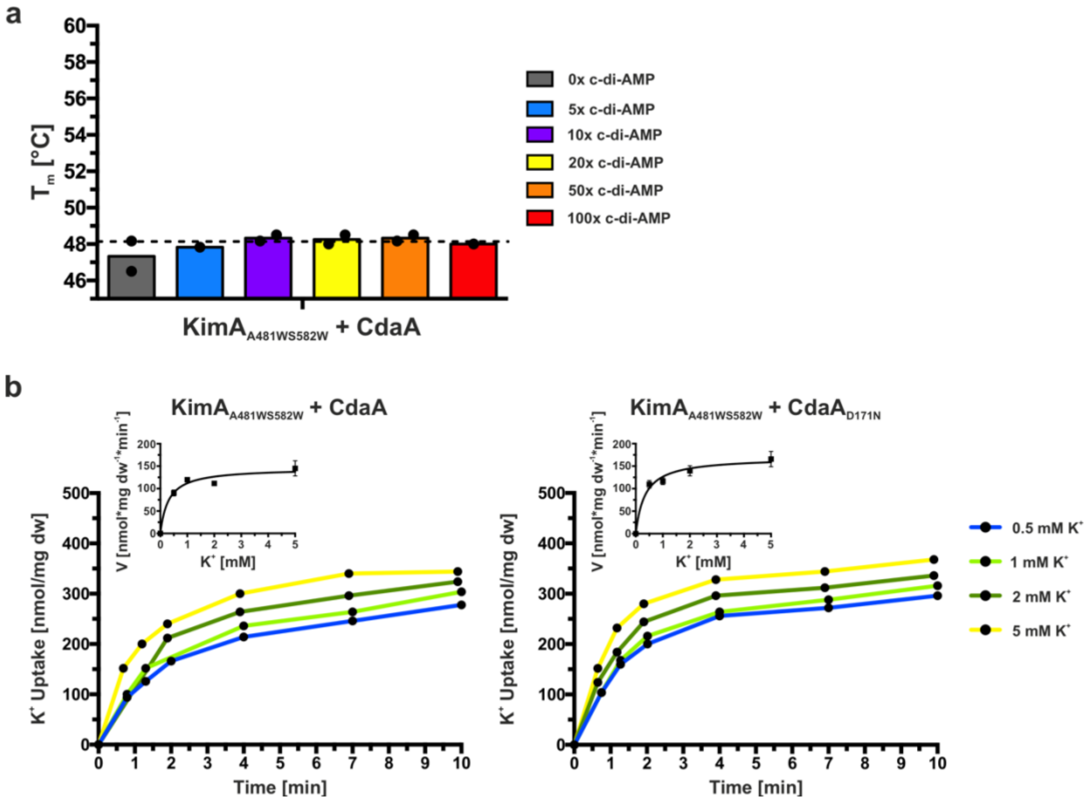


**Supplementary Figure 4: Abolished inhibition by c-di-AMP due to steric blockage of binding pocket in KimA$_{A481WS582W}$ variant. a)** Melting temperature of KimA$_{A481WS582W}$ purified from cells with CdaA present (KimA$_{A481WS582W}$ + CdaA) and incubated with an increasing c-di-AMP concentration given in x-fold molar excess over KimA. Determined with Differential Scanning Fluorometry (DSF). Dashed line indicates $T_m$ of KimA WT w/o c-di-AMP addition. Data points represent the average of measurements from a biological duplicate. **b)** Potassium uptake of *E. coli* LB2003 cells expressing *kimA$_{A481WS582W}$* and *cdaA*/*cdaA$_{D171N}$* at increasing external potassium concentrations. One representative experiment shown (n= 4/4). Michaelis-Menten diagram included in graph with mean value ± SER.

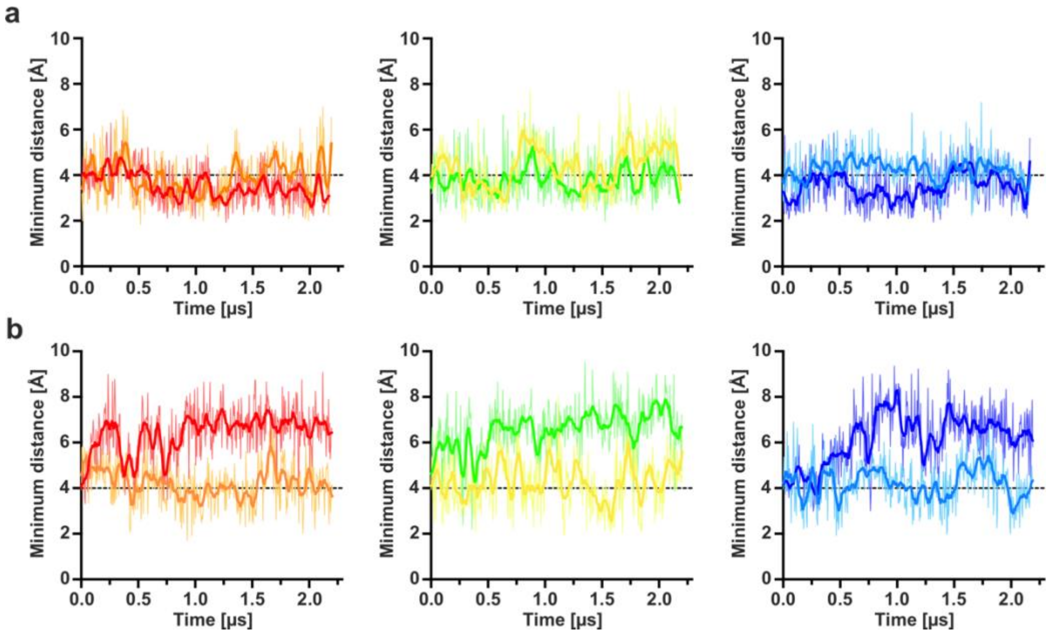


**Supplementary Figure 5: Minimum distance plots between Tyr118 and Asn237 with ligand (a) and without ligand (b).** Each graph is for one simulation, with the two traces representing the two KimA monomers. A moving average is plotted for each dataset, using 10 neighbors and a second order polynomial. A dotted line at 4 Å denotes the approximate cutoff for whether the residues are in contact.

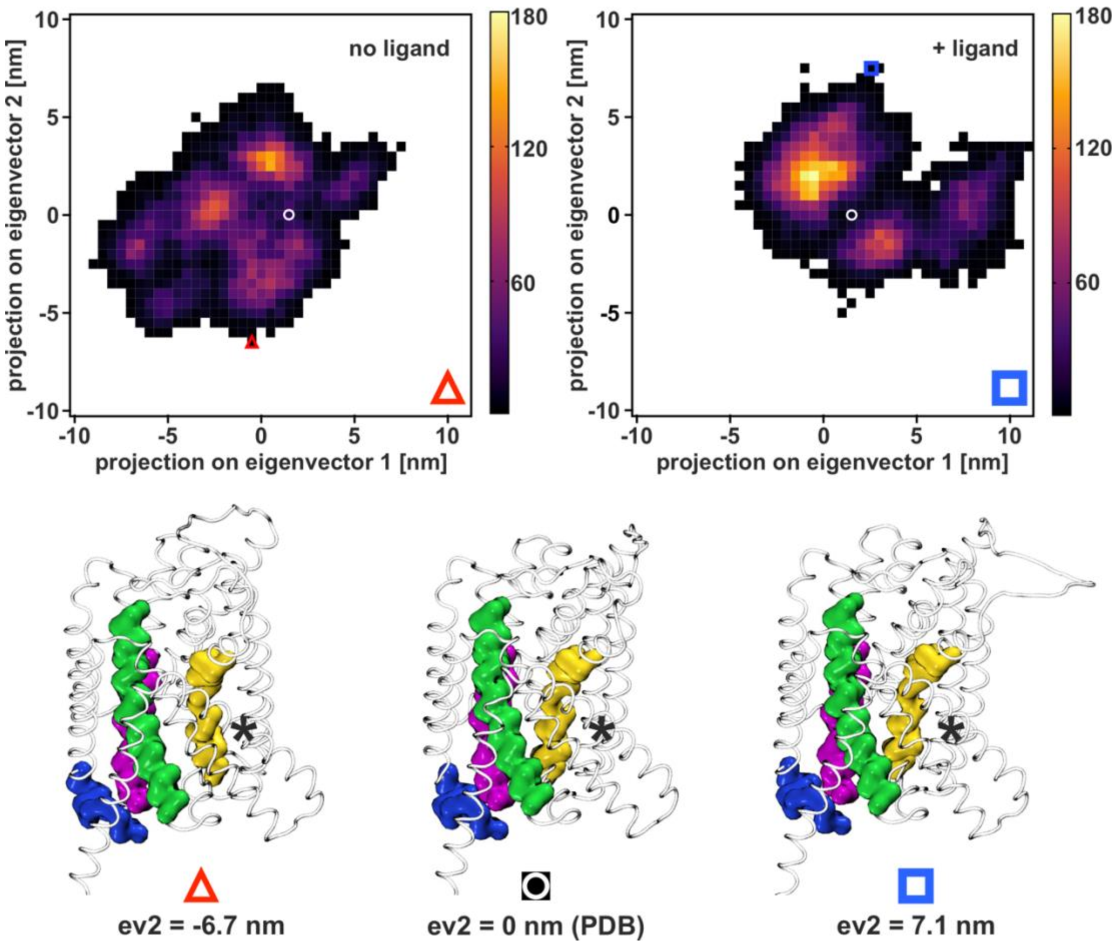


**Supplementary Figure 6**: **Eigenvector 2 PCA analysis.** As Figure 5c, but showing the extreme structural states for eigenvector 2 on the bottom. As in eigenvector 1, in eigenvector 2 there is movement of TM6 (yellow) away from TM3/8 in the non-liganded state (compare asterisks).

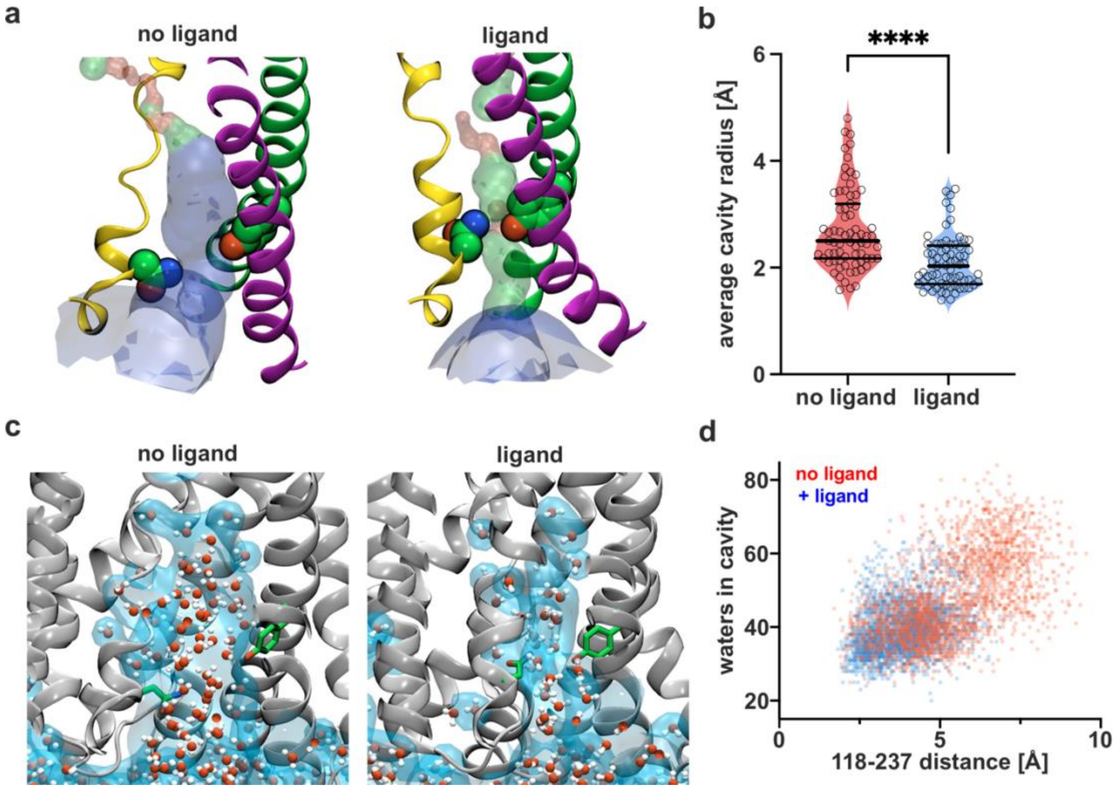


**Supplementary Figure 7**: **Analyses of the cavity in the ligand bound and ligand free KimA states**. **(a)** View of post 2.2 µs snapshots of KimA with no ligand or with c-di-AMP bound. TMs 3, 6 and 8 are shown as green, yellow and purple helices. Tyr 118 and Asn 237 are shown as spheres. HOLE analysis was run on the proteins HOLE[1] which reveals a wider cavity in the ligand free state. **(b)** Quantification of cavities using HOLE on snapshots taken after 1 µs every 50 ns. 75 snapshots were analysed for each ligand state, using the COM between Tyr 118 and Asn 237 as in initial seeding point. To obtain an estimate of cavity size, average radii were computed for the 0.75 nm on either side of the starting position. Violin plots show the median and upper/lower quartiles. Two-tailed t-tests between the data demonstrate significance ($p < 0.0001$). **(c)** Snapshots of post 2.2 µs snapshots of KimA with no ligand or with c-di-AMP bound, using the same poses as panel a. Shown are water molecules in the cavity, suggesting a higher degree of solvation in the no ligand state. **(d)** Plot of the number of solvent molecules in each KimA inward facing cavity vs distance of Tyr 118 and Asn 237 (data from Supplementary Figure 4). Solvent count taken as number of molecules within 1.2 nm of Thr121, which is at the top of the cavity, see Figure 2b.

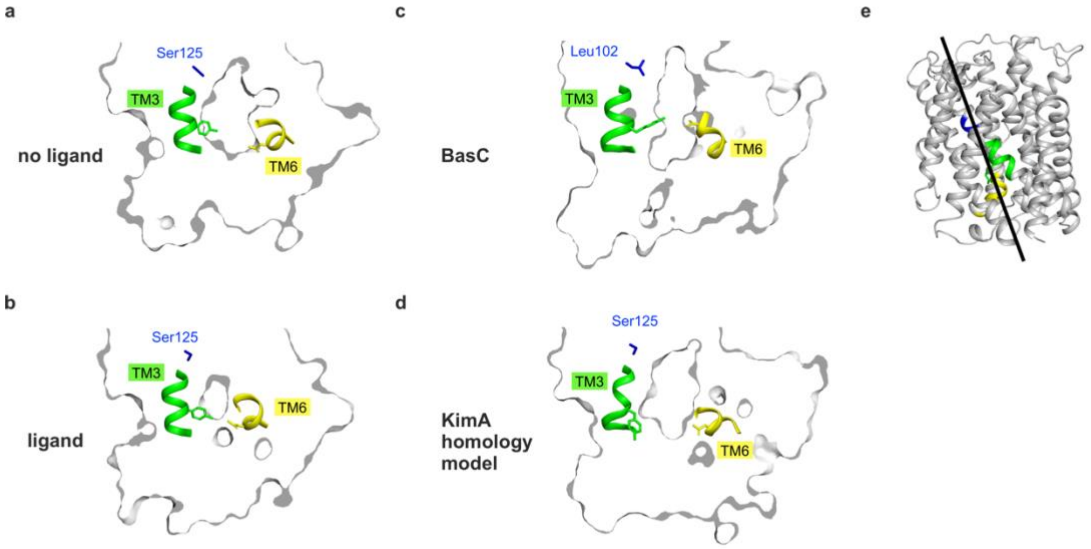


**Supplementary Figure 8**: **Comparison of KimA states to BasC.** Views of KimA post 2.2 µs simulation; snapshot with **(a)** no ligand or **(b)** with ligand bound. The protein is slabbed heavily, as per panel e, to show the size of the cavity between TM3 and TM6. Ser 125 at the top of the cavity is shown for context. **(c, d)** As panels (a) and (b), but for **(c)** BasC in an inward-open conformation (PDB 6F2W[2]) and **(d)** a KimA homology model built on that BasC structure. **(e)** Sideways view of KimA showing position of slice used for panels **a** and **b**.

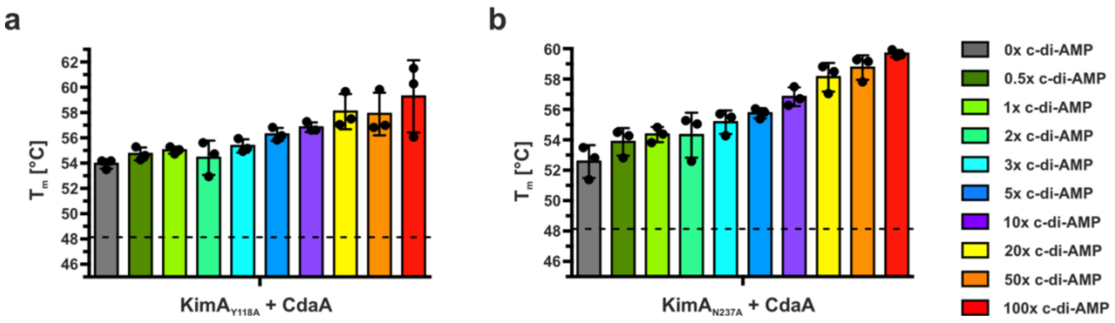


**Supplementary Figure 9: Melting temperature of KimA$_{Y118A}$ + CdaA (a) and KimA$_{N237A}$ + CdaA (b).** Melting temperatures of KimA variants purified from cells with CdaA and incubated with an increasing c-di-AMP concentration given in x-fold molar excess over KimA. Determined with Differential Scanning Fluorometry (DSF). Dashed line indicates $T_m$ of KimA WT w/o c di AMP addition. Data points represent the average and error bars the standard deviation of measurements from biological triplicates.

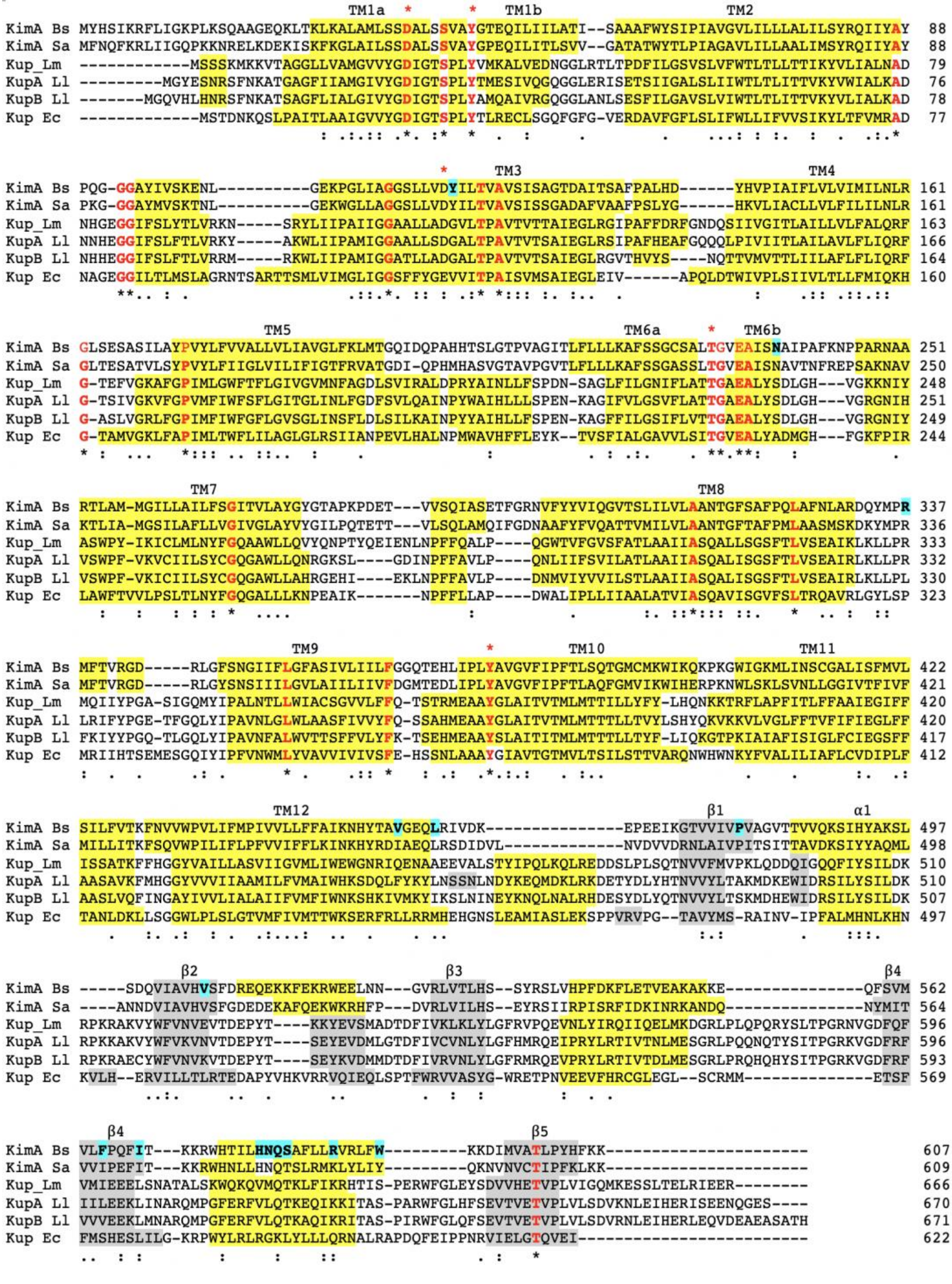


**Supplementary Figure 10: Structure-based alignment of KUPs.** AlphaFold predictions of KimA from *S. aureus* (KimA Sa), Kup from *L. monocytogenes* (Kup Lm), KupA and KupB from *L. lactis* IL1403 (KupA Ll and KupB Ll), and Kup from *E. coli* (Kup Ec) were aligned to the *B. subtilis* KimA structure (KimA Bs). Conserved residues are in bold red font, α-helices are highlighted in yellow and β-strands in grey. TMHs and β-strands in *B. subtilis* KimA are numbered. Residues involved in potassium binding are indicated with a red asterisk. Residues implicated in c-di-AMP binding and inhibition (see text) are highlighted in cyan.

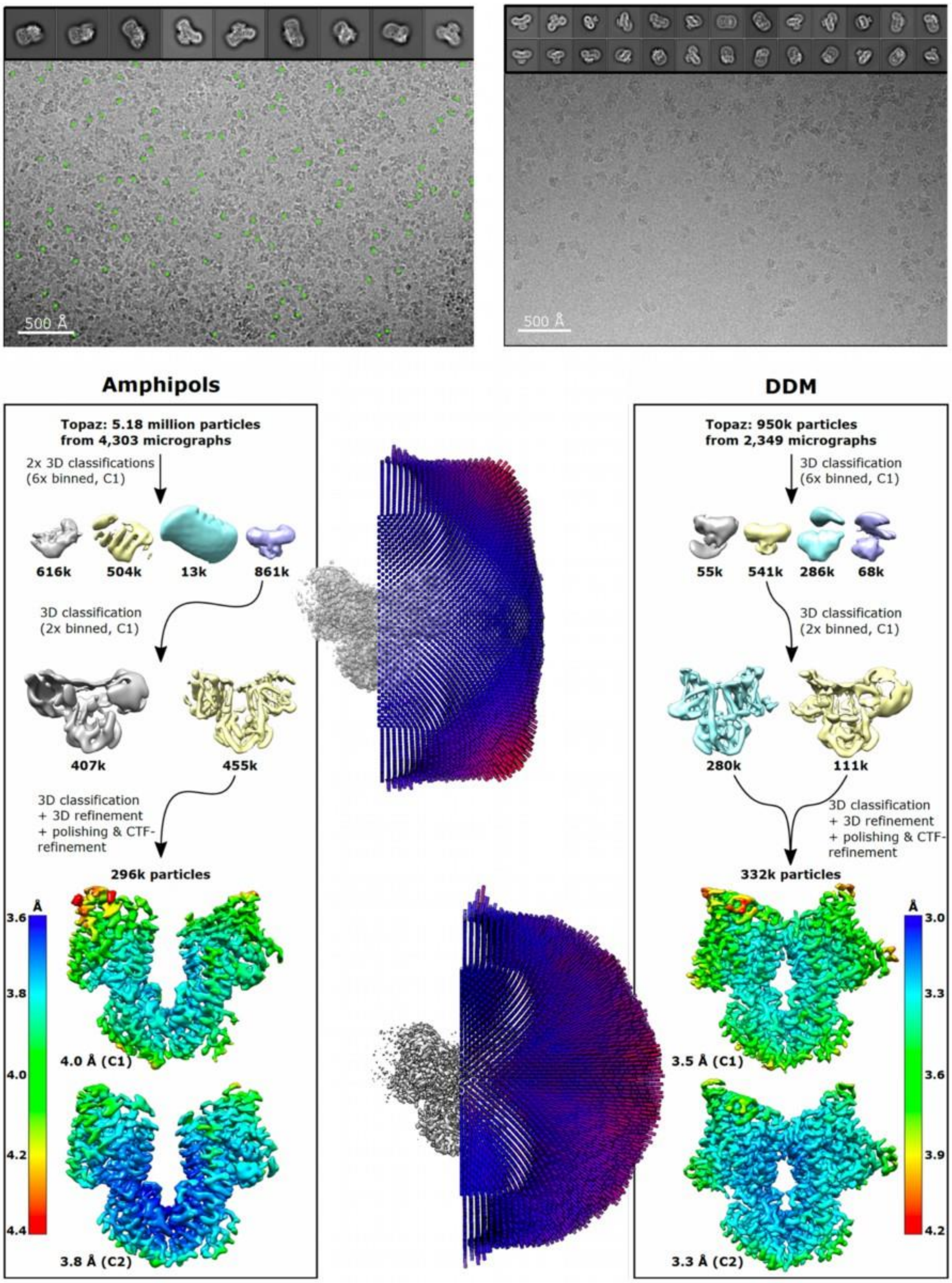


**Supplementary Figure 11:** Exemplary cryo-electron micrographs of KimA reconstituted in amphipols (left, best picks marked green) and in DDM with a ten-fold excess of c-di-AMP (right) respectively, with best 2D class averages, and cryo-EM processing workflows. Centre: angular distribution of particles.

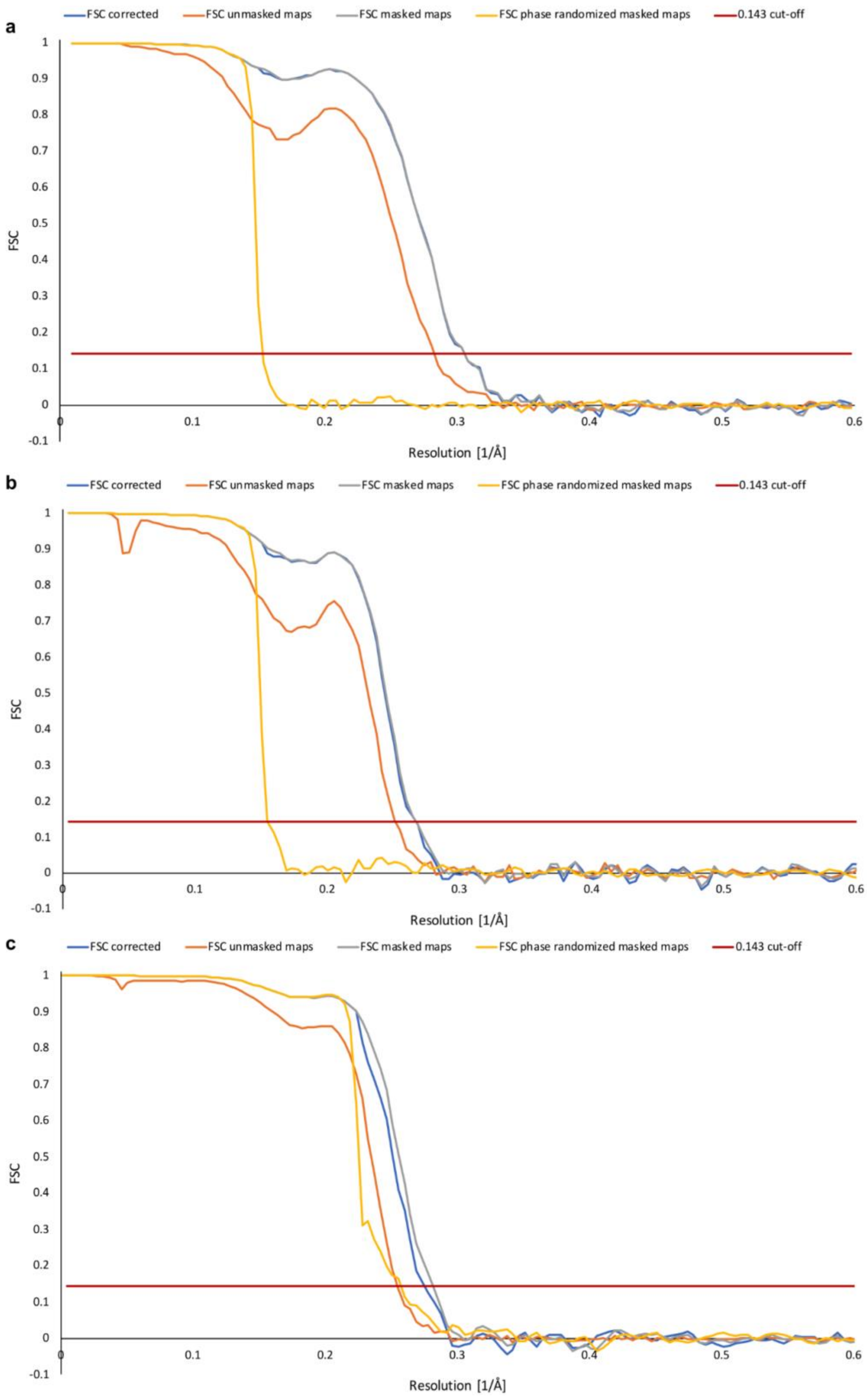


**Supplementary Figure 12:** FSCs of final maps of KimA with bound c-di-AMP, determined by the postprocessing procedure in Relion. **a)** C2 refinement of 332k particles of KimA solubilized in DDM with ten-fold excess of c-di-AMP added after purification. **b)** 296k particles of KimA in amphipols with C2 symmetry applied during refinement. **c)** 307k C2-symmetry expanded particles (KimA in amphipols) after focused 3D-classification and 3D-refinement focused on one half of the dimer.

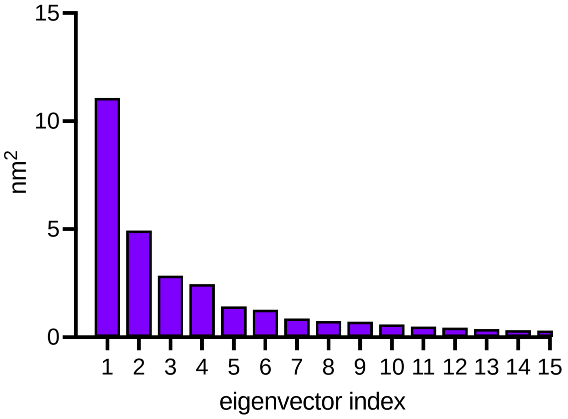


**Supplementary Figure 13: Contribution of each eigenvector to the total variance ($nm^2$).** Only the top 15 are shown out of ca. 1700. Eigenvector 1 and 2 account for ca. 46% of the total variance.

**Supplementary Table 1: Primer used for site-directed mutagenesis in pB24*kimA*.**

| **Primer name** | **Sequence 3'[illegible]5'** |
|---|---|
| **KimA R337A Fwd** | CCAGTATATGCCGGCAATGTTTACAGTCAG |
| **KimA R337A Rev** | CCCTGACTGTAAACATTGCCGGCATATACT |
| **KimA A481W Fwd** | GTTGTGATTGTGCCTGTGTGGGGTGTCACCACCG |
| **KimA A481W Rev** | CGGTGGTGACACCCCACACAGGCACAATCACAAC |
| **KimA S582W Fwd** | CCTTCACAACCAATGGGCCTTCCTCCTCAGAG |
| **KimA S582W Rev** | CTCTGAGGAGGAAGGCCCATTGGTTGTGAAGG |
| **KimA Y118A Fwd** | GCTTGTTGATGCTATTTTAACAGTAG |
| **KimA Y118A Rev** | CTACTGTTAAAATAGCATCAACAAGC |
| **KimA N237A Fwd** | GAGGCCATTTCTGCTGCGATTCCTG |
| **KimA N237A Rev** | CAGGAATCGCAGCAGAAATGGCCTC |

**Supplementary Table 2: Cryo-EM data collection, refinement and model statistics**

| | **DDM** | **Amphipols** |
|---|---|---|
| **Data collection**<br>Microscope | FEI Titan Krios | FEI Titan Krios |
| Camera | Gatan K3 BioQuantum | Gatan K3 BioQuantum |
| Voltage (kV)<br>Nominal magnification<br>Calibrated pixel size (Å) | 300<br>105,000x<br>0.837 | 300<br>105,000x<br>0.837 |
| Electron exposure ($e^-$/Å$^2$) | 55.0 | 55.0 |
| Exposure time total (s)<br>Number of frames per image | 2<br>50 | 2<br>50 |
| Defocus range (µm) | -1.1 – -2.5 | -1.1 – -2.5 |
| **Image processing**<br>Motion correction software | *Relion* | *Relion* |
| CTF estimation software | *Gctf* | *Gctf* |
| Particle selection software | *Topaz* | *Topaz* |
| Micrographs (no.) | 2,349 | 4,303 |
| Initial particle images (no.) | 950,000 | 5,180,000 |
| Final particle images (no.) | 332,00 | 296,000 |
| Symmetry | C2 | C2 |
| Final resolution (Å) | 3.3 | 3.8 |
| **Refinement statistics**<br>Modeling software | *COOT, PHENIX* | *COOT, PHENIX* |
| Protein residues | 572 | 552 |
| Ligands | 2 c-di-AMP, 2 $K^+$,<br>8 DDM | 2 c-di-AMP |
| Map CC (volume) | 0.86 | 0.73 |
| RMS deviations<br>Bond lengths (Å) | 0.006 | 0.005 |
| Bond angles (°) | 0.740 | 0.613 |
| Average B-factor (Å$^2$) | 119.16 | 19.79 |
| Ramachandran plot<br>Outliers (%) | 0.0 | 0.0 |
| Allowed (%) | 5.0 | 5.0 |
| Favored (%) | 95.0 | 95.0 |
| Rotamer outliers (%) | 1.0 | 0.4 |
| Molprobity score | 1.77 | 1.95 |
| All-atom clashscore | 8 | 12 |

**Supplementary Videos**

V1: Cooperativity Network mobility of CDs in the absence of c-di-AMP. Movie showing the dynamics of selected residues in the KimA cytosolic domain in the absence of c-di-AMP. Movie made over a single 2.2 µs simulation trajectory using VMD, region viewed as in figure 3a.

V2: Cooperativity Network mobility of CDs in the presence of c-di-AMP. Movie showing the dynamics of selected residues in the KimA cytosolic domain in the presence of two bound c-di-AMP. Movie made over a single 2.2 µs simulation trajectory using VMD, region viewed as in figure 3a.

V3: Cooperativity Network mobility of CDs in the presence of only one. Movie showing the dynamics of selected residues in the KimA cytosolic domain in the presence of only one bound c-di-AMP. Movie made over a single 0.5 µs simulation trajectory using VMD, region viewed as in figure 3a.

**References**

1. Smart, O. S., Neduvelil, J. G., Wang, X., Wallace, B. A. & Sansom, M. S. P. HOLE: A program for the analysis of the pore dimensions of ion channel structural models. *J Mol Graph* **14**, 354–360 (1996).
2. Errasti-Murugarren, E. *et al.* L amino acid transporter structure and molecular bases for the asymmetry of substrate interaction. *Nat Commun* **10**, 1807 (2019).
