# Supplementary material for: Cyclic di-AMP traps proton-coupled K+ transporters of the KUP family in an inward-occluded conformation
Source: Nat Commun. 2023 Jun 21;14:3683. doi: 10.1038/s41467-023-38944-1 (PMC10284832; doi:10.1038/s41467-023-38944-1)
Supplement: Supplementary file 3 — Description of Additional Supplementary Files [file 41467_2023_38944_MOESM3_ESM.pdf]

Description of Additional Supplementary Files

File Name: Supplementary Movie 1
Description: Cooperativity Network mobility of CDs in the absence of c-di-AMP. Movie showing the dynamics of selected residues in the KimA cytosolic domain in the absence of c-di-AMP. Movie made over a single 2.2 μs simulation trajectory using VMD, region viewed as in figure 3a.

File Name: Supplementary Movie 2
Description: Cooperativity Network mobility of CDs in the presence of c-di-AMP. Movie showing the dynamics of selected residues in the KimA cytosolic domain in the presence of two bound c-diAMP. Movie made over a single 2.2 μs simulation trajectory using VMD, region viewed as in figure 3a.

File Name: Supplementary Movie 3
Description: Cooperativity Network mobility of CDs in the presence of only one. Movie showing the dynamics of selected residues in the KimA cytosolic domain in the presence of only one bound c-diAMP. Movie made over a single 0.5 μs simulation trajectory using VMD, region viewed as in figure 3a.
